# Risk factors for stunting among children under five years: a cross-sectional population-based study in Rwanda using the 2015 Demographic and Health Survey

**DOI:** 10.1186/s12889-019-6504-z

**Published:** 2019-02-11

**Authors:** Alphonse Nshimyiryo, Bethany Hedt-Gauthier, Christine Mutaganzwa, Catherine M. Kirk, Kathryn Beck, Albert Ndayisaba, Joel Mubiligi, Fredrick Kateera, Ziad El-Khatib

**Affiliations:** 1 Partners In Health / Inshuti Mu Buzima, Kigali, Rwanda; 2 Department of Global Health and Social Medicine, Harvard Medical School, Boston, USA; 3 Department of Biostatistics, Harvard T.H. Chan School of Public Health, Boston, USA; 4 Department of Public Health Sciences, Karolinska Institutet, Stockholm, Sweden; 5 World Health Programme, Université du Québec en Abitibi-Témiscamingue (UQAT), Québec, Canada

**Keywords:** Child growth stunting, Child malnutrition, Sub-Saharan Africa, Rwanda

## Abstract

**Background:**

Child growth stunting remains a challenge in sub-Saharan Africa, where 34% of children under 5 years are stunted, and causing detrimental impact at individual and societal levels. Identifying risk factors to stunting is key to developing proper interventions. This study aimed at identifying risk factors of stunting in Rwanda.

**Methods:**

We used data from the Rwanda Demographic and Health Survey (DHS) 2014–2015. Association between children’s characteristics and stunting was assessed using logistic regression analysis.

**Results:**

A total of 3594 under 5 years were included; where 51% of them were boys. The prevalence of stunting was 38% (95% CI: 35.92–39.52) for all children. In adjusted analysis, the following factors were significant: boys (OR 1.51; 95% CI 1.25–1.82), children ages 6–23 months (OR 4.91; 95% CI 3.16–7.62) and children ages 24–59 months (OR 6.34; 95% CI 4.07–9.89) compared to ages 0–6 months, low birth weight (OR 2.12; 95% CI 1.39–3.23), low maternal height (OR 3.27; 95% CI 1.89–5.64), primary education for mothers (OR 1.71; 95% CI 1.25–2.34), illiterate mothers (OR 2.00; 95% CI 1.37–2.92), history of not taking deworming medicine during pregnancy (OR 1.29; 95%CI 1.09–1.53), poorest households (OR 1.45; 95% CI 1.12–1.86; and OR 1.82; 95%CI 1.45–2.29 respectively).

**Conclusion:**

Family-level factors are major drivers of children’s growth stunting in Rwanda. Interventions to improve the nutrition of pregnant and lactating women so as to prevent low birth weight babies, reduce poverty, promote girls’ education and intervene early in cases of malnutrition are needed.

## Background

In 2016, an estimated 155 million children worldwide were stunted (defined as being too short for their age); over one-third of them resided in Africa [1, 2]. Currently, in sub-Saharan Africa (SSA), 34% of children aged less than five years are stunted and the burden of stunting is most prevalent in the Eastern African region (37% stunted) [2]. Growth stunting is considered an important indicator of child health inequalities [3, 4]. It can be caused by several factors in both pre- and post-natal phases of development, some of these factors include poor nutrition, infectious diseases, and household environment [5]. A global review of stunting in low- and middle-income countries identified growth restriction in-utero and lack of access to sanitation as the main drivers of stunting [6]. Thus early interventions are necessary to prevent stunting; after the first two years of life, stunting is often irreversible [7].

Several studies have demonstrated the negative and long-term impacts of stunting on early childhood development such as poor performance at school and low productivity when they reach their working age [8, 9]. Stunted children remain behind their non-stunted peers later in life [10]. Rwanda lost over US$48 million of its 2012 GDP from an estimated three million people (49%) of working age (16–64 years old) who suffered from stunting as children [11]. Repetition of grades and school dropout rates among stunted children were also associated with significant economic losses for the education system, families, and the labor market. Stimulation interventions can improve cognitive outcomes in children who are stunted; however, these interventions are insufficient for eliminating the negative effects of stunting [10].

More than one in five child deaths were attributed to under-nutrition in 2012 [11]. Hence, the government of Rwanda has developed a plan to eliminate all forms of malnutrition with a special focus on growth stunting [12, 13]. Under this plan, numerous interventions to improve maternal and child nutrition have been implemented, e.g., micronutrient supplementation for both the mother and her child, deworming, a nationwide campaign on the first 1000 days of a child’s life to change behaviors around maternal care and maternal and child nutrition, timing and appropriate diet for complementary feeding, child care and hygiene [14]. While the proportion of stunted Rwandan children decreased between 2005 and 2015 (from 51 to 38%) [15], growth stunting is still considered a major challenge to overall economic development in Rwanda, particularly among the poor children (49% stunted) and children living in rural areas (41% stunted) [15, 16]. To better understand the persisting challenges to reducing stunting in Rwanda, we used the 2014–2015 Demographic and Health Survey (DHS) data and conducted an assessment for 17 risk factors for stunting across all provinces in Rwanda, based on the World Health Organization (WHO) conceptual framework for childhood stunting [17, 18].

## Methods

### Data sources

We used the 2014–2015 Rwanda DHS open access dataset [19]. The DHS included a randomly selected national total of 12,793 households from five provinces. Of the 12,793 households, a sub-sample of 6350 (50%) households was randomly selected and data on child anthropometric measurements and development indicators were collected (*N* = 3594 children) [15]. A full protocol explaining the data collection process and sampling methods of DHS can be reviewed elsewhere [19].

### Variables description

We have included a total of 17 variables related to three categories in the WHO stunting framework [17, 18]: i) Individual-level factors (sex, age group, parity, child’s weight at birth, history of diarrhea in two weeks prior to the survey); ii) Maternal (height, highest educational level, intake of parasite controlling drugs for mothers during pregnancy, number of days of daily intake of iron tablets or syrup by mothers during pregnancy, breastfeeding within the first hour after birth and household (household wealth index, household size, access to improved water at household, availability of improved toilet facility) factors and iii) Community level factors (household location data including province, sector and village; altitude (highland vs. lowland) and location (urban vs. rural).

### Definitions

*Stunting* was measured by DHS, using the WHO Child Growth Standards and collected data on every child’s length/height, age and sex to calculate the number of standard deviations (Z-score) that his/her length/height is below or above the median of the 2006 WHO growth reference population [20]. These measures were recorded with two implied decimal places, thus we divided all values of DHS standard deviations by a hundred. Stunting was defined as a z-score lower than − 2. *Mother’s height* was considered low if it was < 145 cm [21]. *Household wealth index* was calculated using principal components analysis (PCA) of data on a household’s ownership of assets like television, radio, mobile telephone, computer and car, and housing characteristics (access to electricity, access to source of drinking water, type of floor material, type of toilet facility, number of bedrooms, and type of cooking fuel) [15]. Wealth index scores were adjusted for the type of residence (rural or urban). Then, DHS assigned each person in the population his/her household’s score. The study population was ranked according to their social economic scores and divided into five equal groups (20% of the population), known as wealth quintiles [22]. For this study, we re-grouped our sample of children into three groups: High-social economic status (SES) (highest two wealth quintiles), middle SES (middle wealth quintile) and low-SES (lowest two wealth quintiles) [15].

*Child weight at birth* was defined as low if it was less than 2.5 kg [23].

*Location of the household* was defined as highland if the household location is at an altitude of > 1642 m (median altitude of households), and lowland if the household location is at an altitude of < 1642 m.

#### Data analysis

To determine risk factors for stunting, we first conducted a full logistic regression model with all 17 variables by calculating odds ratio (OR), 95% confidence interval (CI) and *p*-value. Then, we conducted a final logistic regression model by controlling for sex, province, and altitude. All variables with *p*-values 0.10 or less in the full model were considered in our final logistic regression model, and removed using backward stepwise selection stopping when all the final variables were significant at the α = 0.05 significance level. Stata/SE 13.1 was used for data analysis [24]. We used the *svy* commands to account for the complex survey sampling and used sampling weights to account for unequal probability sampling in different strata.

## Results

### Descriptive analysis

A total of 3594 children were included in this study (Table 1). Almost half of these children were females (49%; 1769/3594). At birth, only 5% (173/3329) of the children had low weight. Over half (58%; 2091/3594) of the children were 24–59 months of age, and 72% (2551/3565) had access to improved source of drinking water in the household. As for geographical location, 11% (406/3593) lived in the capital city of Kigali, 14% (500/3593) in the Northern Province and the rest were almost equally distributed among the three remaining provinces. Slightly less than half of them (48%; 1728/3594) lived in highlands, and 84% (3003/3594) in rural areas.

**Table 1:** Socio-demographic and economic characteristics of children 0-59 months, Demographic and Health Survey (DHS), Rwanda, 2014-15

Characteristics	Total		Stunting				
			Yes		No		*p* value
	N	(%)	N	(%)	N	(%)	
Individual-level factors							
Child sex							
Females	1769	(49%)	581	(33%)	1188	(67%)	
Males	1825	(51%)	774	(42%)	1051	(58%)	< 0.01
Child’s age group							
< 6 months	332	(9%)	35	(11%)	297	(89%)	
6–23 months	1171	(33%)	425	(36%)	745	(64%)	
24–59 months	2091	(58%)	894	(43%)	1197	(57%)	< 0.01
Child parity - First child							
Yes	995	(28%)	345	(35%)	650	(65%)	
No	2598	(72%)	1009	(39%)	1589	(61%)	0.03
Child weight at birth							
≥ 2.5 Kg	3156	(95%)	1131	(36%)	2025	(64%)	
< 2.5 Kg	173	(5%)	94	(54%)	79	(46%)	< 0.01
History of diarrhea - two weeks prior to the survey							
No	3131	(87%)	1147	(37%)	1984	(63%)	
Yes	462	(13%)	207	(45%)	255	(55%)	< 0.01
Maternal/household factors							
Mother height (cm)							
≥ 145 cm	3506	(98%)	1296	(37%)	2210	(63%)	
< 145 cm	88	(2%)	59	(67%)	29	(33%)	< 0.01
Mother’s highest educational level							
Secondary/higher	463	(13%)	90	(19%)	373	(81%)	
Primary	2615	(73%)	1021	(39%)	1594	(61%)	
No education	516	(14%)	245	(47%)	271	(53%)	< 0.01
History of intake of parasite drugs for mothers during pregnancy							
Yes	1374	(50%)	449	(33%)	925	(67%)	
No	1396	(50%)	547	(39%)	849	(61%)	< 0.01
Duration of intake of daily iron tablets or syrup by mothers during pregnancy							
≥ 30 days	1532	(71%)	531	(35%)	1001	(65%)	
0–29 days	621	(29%)	250	(40%)	371	(60%)	0.01
Breastfeeding within the first hour after birth							
Yes	2839	(79%)	1046	(37%)	1793	(63%)	
No	742	(21%)	304	(41%)	439	(59%)	0.06
Household wealth index							
High (rich)	1211	(34%)	299	(25%)	912	(75%)	
Middle	700	(19%)	264	(38%)	436	(62%)	
Low (Poor)	1682	(47%)	791	(47%)	891	(53%)	< 0.01
Number of household members							
5 people or less	2176	(61%)	826	(38%)	1350	(62%)	
> 5	1417	(39%)	529	(37%)	888	(63%)	0.73
Availability of improved water at household							
Yes	2551	(72%)	902	(35%)	1649	(65%)	
No	1014	(28%)	444	(44%)	570	(56%)	< 0.01
Toilet (Sanitation facility)							
Improved	2603	(73%)	945	(36%)	1658	(64%)	
Not improved	958	(27%)	400	(42%)	558	(58%)	< 0.01
Community level factors							
Province of residence							
Kigali City	406	(11%)	92	(23%)	314	(77%)	
South	853	(24%)	347	(41%)	506	(59%)	
West	856	(24%)	381	(45%)	475	(55%)	
North	500	(14%)	194	(39%)	306	(61%)	
East	978	(27%)	341	(35%)	637	(65%)	< 0.01
Household location - Altitude							
Lowland (< 1641.5 m)	1866	(52%)	606	(32%)	1260	(68%)	
Highland (≥1641.5 m)	1728	(48%)	749	(43%)	979	(57%)	< 0.01
Household type of residence							
Urban	591	(16%)	140	(24%)	451	(76%)	
Rural	3003	(84%)	1215	(40%)	1788	(60%)	< 0.01

The prevalence of stunting was 38% (95% CI: 35.92, 39.52) in the sample. Over half of stunted children were males (57%; 774/1355; *p* < 0.01); 66% (894/1355) of them were aged 24–59 months (*p* < 0.01), and 55% (749/1355) lived in highland areas (*p* < 0.01). Most of children with stunting (79%; 1069/1355) were residents of the Western, Southern and Eastern provinces.

The median age of the mothers was 30 years (Interquartile range (IQR) 26–34), and most of them had at least primary school education (73%, 2615/3593). Over one-quarter of women (28%, 995/3593) had only one child at the time of the survey. When it comes to the history of antenatal medication intake, 45% (449/996) and 68% (531/781) of the mothers of stunted children took an anti-parasitic drug and at least 30 tablets of iron supplementation respectively, during pregnancy.

#### Risk factors for growth stunting

In the full logistic regression model, factors associated with growth stunting were: a) Individual characteristics, including higher risk among males (OR 1.61, 95% CI 1.30–1.99, *p* < 0.01), children aged 6–23 months (OR 4.43, 95% CI 2.74–7.16, *p* < 0.01) and 24–59 months (OR 5.40, 95% CI 3.33–8.76, *p* < 0.01), low child weight at birth (OR 2.00, 95% CI 1.24–3.23, *p* = 0.01); b) Maternal and household characteristics, including mothers’ height < 145 cm (OR 2.81, 95% CI 1.56–5.08, *p* < 0.01), mother’s educational level of either only primary (OR 1.49, 95% CI 1.03–2.15, *p* = 0.04) or never attended school (OR 1.83, 95% CI 1.18–1.61, *p* = 0.01), household middle wealth index (OR 1.36, 95% CI 1.00–1.85, *p* = 0.05) and poorest level (OR 1.98, 95% CI 1.49–2.63, *p* < 0.01) (Table 2).

**Table 2:** Risk factors for stunting among children 0-59 months, Demographic and Health Survey, Rwanda, 2014-15

Characteristics	Full logistic regression model		Final logistic regression model^a^	
	OR (95% CI)	*p* value	OR (95% CI)	*p* value
Individual-level factors				
Child sex				
Females	Ref		Ref	
Males	1.61 (1.30–1.99)	< 0.01	1.51 (1.25–1.82)	< 0.01
Child’s age group				
< 6 months	Ref		Ref	
6–23 months	4.43 (2.74–7.16)	< 0.01	4.91 (3.16–7.62)	< 0.01
24–59 months	5.40 (3.33–8.76)	< 0.01	6.34 (4.07–9.89)	< 0.01
Child parity - First child				
Yes	Ref			
No	1.07 (0.83–1.38)	0.62		
Child weight at birth				
> 2.5 Kg	Ref		Ref	
< 2.5 Kg	2.00 (1.24–3.23)	0.01	2.12 (1.39–3.23)	< 0.01
History of diarrhea in two weeks prior to the survey				
No	Ref			
Yes	1.14 (0.85–1.52)	0.38		
Maternal/household factors				
Mother height (cm)				
≥145 cm	Ref		Ref	
< 145 cm	2.81 (1.56–5.08)	< 0.01	3.27 (1.89–5.64)	< 0.01
Mother’s highest educational level				
Secondary or higher	Ref		Ref	
Primary	1.49 (1.03–2.15)	0.04	1.71 (1.25–2.34)	< 0.01
No education	1.83 (1.18–2.85)	0.01	2.00 (1.37–2.92)	< 0.01
Intake of parasite drugs for mothers during pregnancy				
Yes	Ref		Ref	
No	1.31 (1.06–1.61)	0.01	1.29 (1.09–1.53)	< 0.01
Duration of intake of daily iron tablets or syrup by mothers during pregnancy				
≥30 days	Ref			
0–29 days	1.17 (0.93–1.46)	0.17		
Breastfeeding within the first hour after birth				
Yes	Ref			
No	1.27 (0.96–1.67)	0.09		
Household wealth index				
High (rich)	Ref		Ref	
Middle	1.36 (1.00–1.85)	0.05	1.45 (1.12–1.86)	< 0.01
Low (poor)	1.98 (1.49–2.63)	< 0.01	1.82 (1.45–2.29)	< 0.01
Household size (number of household members)				
5 people or less	Ref			
> 5	1.01 (0.82–1.25)	0.78		
Access to improved water at household				
Yes	Ref			
No	1.19 (0.94–1.51)	0.15		
Toilet (Sanitation facility)				
Improved	Ref			
Not improved	0.91 (0.72–1.15)	0.44		
Community level characteristics				
Province of residence				
Kigali City	Ref		Ref	
South	1.27 (0.79–2.04)	0.32	1.26 (0.84–1.90)	0.26
West	1.24 (0.75–2.04)	0.39	1.34 (0.88–2.05)	0.17
North	1.15 (0.69–1.91)	0.59	1.11 (0.72–1.73)	0.49
East	1.04 (0.66–1.64)	0.86	1.14 (0.79–1.64)	0.69
Household location - Altitude				
Lowland (< 1641.5 m)	Ref		Ref	
Highland (≥1641.5 m)	1.22 (0.92–1.63)	0.17	1.29 (0.99–1.67)	0.05
Household type of residence				
Urban	Ref			
Rural	1.18 (0.86–1.62)	0.29		

^a^Sex, province and altitude were forced into the final model regardless of OR results in the full model

Final model excluded variables with p value > 0.10

In the final logistic regression model, factors associated with growth stunting were: a) Individual characteristics, including higher risk among males (OR 1.51, 95% CI 1.25–1.82, *p* < 0.01), children aged 6–23 months (OR 4.91, 95% CI 3.16–7.62, *p* < 0.01) and 24–59 months (OR 6.34, 95% CI 4.07–9.89, *p* < 0.01), low child weight at birth (OR 2.12, 95% CI 1.39–3.23, *p* < 0.01); b) Maternal and household characteristics, including mothers’ height < 145 cm (OR 3.27, 95% CI 1.89–5.64, *p* < 0.01), mother’s educational level of either only primary (OR 1.71, 95% CI 1.25–2.34) or never attended school (OR 2.00, 95% CI 1.37–2.92, *p* < 0.01), history of not taking anti-parasite drugs during pregnancy (OR 1.29, 95% CI 1.09–1.53, *p* < 0.01), household wealth index middle (OR 1.45, 95% CI 1.12–1.86) and poorest level (OR 1.82, 95% CI 1.45–2.29, p < 0.01); and c) Community level characteristics including living in highlands (OR 1.29, 0.99–1.67, *p* = 0.05).

## Discussion

In this study, we used nationally representative population-based survey data of Rwanda that included nearly 3600 children aged less than 5 years. The prevalence of stunting has decreased from 51% in 2005 to 38% in 2015 in Rwanda [15]. However, approximately two in every five children are stunted, which indicates that this remains a serious public health challenge.

Boys were at a higher risk for stunting than girls, which is consistent with other studies reporting similar trends from sub-Saharan Africa [25–27]. This might be due to preferences in feeding practices or other types of exposures [25]. The nutritional status might be explained by “biological fragility” because boys are expected to grow at a slightly more rapid rate compared to girls and their growth is perhaps more easily affected by nutritional deficiencies or other disease or exposures [28].

We indicated that increasing age of the child had a significant association with stunting. Children aged 6–23 months were at lower risk of stunting than those in the older age group 24–59 months, which has been reported by other studies [22, 29]. Rates of exclusive breastfeeding during the first six months are high in Rwanda (87%), which may provide a protective effect against stunting at early ages [15]. The gradual increase of stunting among children under-5 years in Rwanda might be due to the inappropriate food supplementation during the weaning period when infants should undergo a transition from exclusive breastfeeding to including complementary foods in their diet [30, 31]. Mothers receive education regarding breastfeeding and transition to complementary feeding through the Community Based Nutrition Program (CBNP) at the village level [32], but this is a self-selecting group who choose to attend these educational sessions, and therefore may miss mothers of children who are malnourished or at-risk for developing malnutrition. Additionally, mothers may prolong the duration of breastfeeding, which is known to be a risk factor for stunting [22, 33, 34], especially if they do not know when to commence giving complementary food or due to poverty-related barriers to adequate complementary feeding. According to studies from Bangladesh, Cambodia, Peru and Nepal, educated mothers, coming from wealthy households, tend to be more aware of the nutritional needs of their children [30, 35–38]. Also, the reverse association between household wealth and stunting has been observed in several studies [37–41]. The wealth of households is a proxy for the purchasing power for food and other nutritional goods needed for the health of the children. Therefore, children in low-socioeconomic status households are less likely to be exposed to good nutrition, which will lead to stunting. However, the recent evidence on a slow economic growth and smaller reduction in stunting as a response to the gross national income (GNI) growth in SSA countries has resulted in an increased international focus on scaling-up maternal and child nutrition-specific interventions [42], as nearly the only way to accelerate stunting reduction, and it is worrying that this would undermine the role of poverty in persisting high rates of stunting and its slow reduction in SSA [43]. Overall, rates of stunting in Rwanda (38%) are similar to rates of poverty (39%) [44]; while poverty is reducing overtime from 45% in 2010, and stunting from 44% in 2010 [45], the association between poverty and stunting in our study indicates that further investments in social protection programs that specifically target households with young children, or women of reproductive age, may be further needed in Rwanda to address the high burden of stunting. Rwanda has adopted pro-poor policies to advance the economic development of the country with an equity approach. As a predominantly rural agrarian society, with high population density, efforts to strengthen the agricultural sector are key to reducing food insecurity and advancing economic development as well as investments in human capital starting with promotion of optimal early childhood development [46]. Specific efforts in Rwanda’s social protection system have targeted precisely this group by focusing on cash for work programs that are more gender and child-sensitive, to ensure women who are pregnant and breastfeeding are not unintentionally excluded from the program [47]. The trend of poverty and stunting reducing at similar rates, provides promise that poverty-reduction initiatives are achieving this goal however the ambitious target to reduce stunting at quicker rates may require further government investment to accelerate poverty reduction, which is stagnating according to the latest measure of poverty at 38% in 2017 [48], and strategic interventions targeting specific risk factors for stunting including households at high altitude, low birth weight births, and appropriate feeding across the early years of life.

These study findings will further contribute to evidence to nutritionists and public health researchers to reshape and design new interventions to reduce the prevalence of stunting in Rwanda, starting with upstream prenatal and maternal factors. Our study showed that children born with a low weight (< 2.5 kg) were more likely to be stunted, in comparison to children with weight ≥ 2.5 kg at birth. This finding is aligned with reports from Pakistan, Mexico, and Nepal [22, 49, 50]. Additionally, we found mother’s height has an association with stunting, which is aligned with the findings of the COHORTS group study in Brazil, Guatemala, India, the Philippines and South Africa [51]. These two predictors are related to maternal factors that require an additional understanding of nutrition and health during pregnancy and addressing intergenerational cycles of malnutrition [52]. These maternal factors require investments by the health sector, to improve the nutrition of women of reproductive age and invest in strategies to prevent low birth weight births, such as improved spacing and quality antenatal care and these priorities are part of the latest government of Rwanda strategies in the health sector [53].

A history of use of deworming drugs during pregnancy was associated with a decreased risk of stunting. This association could be a result of reduced helminths infections during pregnancy and promote better absorption of nutrients by the mother. However, interestingly, there was no statistically significant association between stunting and the availability of improved sanitation and water at the household, which are considered by most of the interventions to improve hygiene and prevent the spread of disease and helminths/parasites. Our findings conflict with other studies on stunting and hygiene. Hien and Hoa found access to clean water and the availability of a hygienic toilet as intermediate risk factors for stunting [54], which might indicate a weak association [29]. However, Danei et.al. found that lack of an improved toilet is a major risk factor for stunting in an international meta-analysis on 137 countries in resource-limited settings [6].

Another risk factor for child growth stunting was among children living in households located in high-altitudes. Rwanda is known by the name of “Land of 1,000 Hills” due to its geographical landscape. Villages can exist on the top of these hills in high-altitudes. While we were surprised that our analysis did not show that living in a rural area is a risk factor, this may be due to the fact that many of these rural populations are also high altitude, which was significant. Living in rural high-altitudes has been associated with higher rates of child stunting in Peru [55]. This might be related to environmental and climate factors, differences in diet due to food availability in highlands versus lowlands, and increased level of physical activity for children living in highlands [55]. This finding warrants further investigation into the association between stunting and living in high-altitudes in Rwanda. Community-based interventions for stunting specifically targeted to rural, high-altitude areas may be one strategy for targeting this high-risk group, especially when it comes to complementary feeding, childhood illnesses preventive interventions, and mothers’ education.

### Study limitations

This study was based on the DHS survey which lacked other additional information that we could have included in the analysis as per the WHO Framework for predictors of stunting [17, 18], such as i) Poor hygiene practices, ii) Contamination of water, and iii) Quality of food intake. In addition, most of children’s data in DHS are self-reported by primary caregivers which is subject to a hidden recall bias, however, the DHS sampling method, and the methods of adjustment for the sampling and weighting suggest a good strengths for this study [56].

## Conclusion

Stunting prevalence is high in Rwanda, with rates seen to increase with an increasing age starting among infants on complementary feeding (> 6 months) and into early childhood. Our study findings suggest that stunting could be reduced if integrated interventions are implemented to improve child and family-level factors that contribute to stunting. Efforts to reduce poverty, improve maternal nutrition for preventing low birth weight babies, increase access to quality and timely antenatal care services and strengthen community-based nutrition activities to promote exclusive breastfeeding up to 6 months and continued breastfeeding up to 24 months with the addition of high quality complementary feeding, would accelerate stunting reduction.

## Abbreviations

CI: Confidence interval
DHS: Demographic and Health Survey
Kg: Kilograms
OR: Odds ratio
PCA: Principal components analysis
SES: Socio-economic status
SSA: Sub-Saharan Africa
WHO: World Health Organization
